# Seed wintering and deterioration characteristics between weedy and cultivated rice

**DOI:** 10.1186/1939-8433-5-21

**Published:** 2012-08-17

**Authors:** Jung-Sun Baek, Nam-Jin Chung

**Affiliations:** Department of Crop Science and Biotechnology, Chonbuk National University, Jeonju, 561-756 Republic of Korea

**Keywords:** Weedy rice, Seed, Wintering, Deterioration, Freezing resistance, Antioxidant, Accelerated aging

## Abstract

**Background:**

Incidences of weedy rice continuously occurred in paddy fields because its shattering seeds were able to over-winter. In this research, the seed deterioration of weedy rice was investigated compared with cultivated rice, and the wintering characteristics of these two types of rice were investigated with the field wintering test, freezing resistance test, and accelerated aging test.

**Results:**

For the wintering test, the seeds of weedy rice were placed on the soil surface of a paddy with cultivated rice seeds during the 2008/2009 and 2009/2010 winter seasons from November to April. The viability of seeds after wintering was 4.3% for cultivated rice, but 92.7% for weedy rice in 2008/2009. In the second wintering test, the seeds were placed under flooded and dry paddy conditions. The seed viability of cultivated rice was 5% in dry paddy and 0.5% in flooded paddy, but weedy rice maintained a high viability during winter of 90% in the dry paddy and 61% in the flooded paddy. Following freezing treatment of the imbibed seeds, the seed viability was 78% for weedy rice and 16% for cultivated rice. The deterioration of seed tissue induced by freezing treatment was observed by the tetrazolium test. In an accelerated aging test at low temperature and soaking conditions, the seed viability of the weedy rice was 40% higher than the cultivated rice 90 days after treatment. During accelerated aging of seeds, the protein content remained higher in the weedy rice compared to the cultivated rice, and fat acidity remained lower in the weedy rice compared to the cultivated rice. Catalase and superoxide dismutase activity of the weedy rice was 4 times higher than that of the cultivated rice, and DPPH radical scavenging activity of the weedy rice was also much higher than for the cultivated rice.

**Conclusion:**

In conclusion, the superior ability of seed wintering in weedy rice was based on freezing resistibility of embryo cellular tissue and higher antioxidant activity to protect seed deterioration during the winter season.

**Electronic supplementary material:**

The online version of this article (doi:10.1186/1939-8433-5-21) contains supplementary material, which is available to authorized users.

## Background

Traditional rice cultivation has been via transplanting of rice seedlings. However, due to the severe problems of agricultural water scarcity and farm labor shortage, direct-seeding methods have been practiced in many parts of the world (Cao et al. [2007]; Savary et al. [2005]; Tabbal et al. [2002]; Tomita et al. [2003]). In most countries, weedy rice infestation increased significantly after shifting from rice transplanting to direct seeding, and it was recognized as a noxious weed (Ottis et al. [2005]; Suh [2003]; Vaughan et al. [2001]). Weedy rice infestations have been reported to have spread to 40–75% of the total area of rice cultivation in Europe, 40% in Brazil, 55% in Senegal, 80% in Cuba, and 60% in Costa Rica (Fogliatto et al. [2010]).

The occurrence of weedy rice has very large effects on rice yield (Pantone and Baker [1991]; Pantone et al. [1992]). Weedy rice infestations are responsible for significant yield losses, which are particularly severe in short varieties and late planting cultivation (Fogliatto et al. [2010]). Weedy rice is so productive that it can spread and cause major economic damage (Kane and Baack [2007]), but it is impossible to control weedy rice during the rice cultivation period by herbicide because it belongs to the same species as the cultivated rice (Delouche et al. [2007]; Suh [2003]).

Weedy rice looks similar to cultivated rice in outward appearance, but has a high level of adapting ability to environmental stress in physiological traits. Being distinguishable by a red pericarp, it disperses immediately by shattering at maturity, guaranteeing its continuation (Chung [2010]; Chung and Ahn [2005]; Chung and Paek [2003]; Ottis et al. [2005]; Suh [2008]). The shattering nature of weedy rice after ripening makes it difficult to be removed when cultivated rice is harvested. The occurrence of weedy rice becomes more severe the following year due to shattered seed present in the soil (Seong et al. [2004]).

The reason of continuous incidences of weedy rice was that its shattering seeds were able to over-winter. In this respect, the deterioration of weedy rice seeds during winter might be different with cultivated rice in freezing resistance and antioxidant activities. There were no reports on the freezing resistance, but some reports of antioxidant activities related with seed deterioration in rice seeds (Bailly [2004]). Generally, it has become increasingly accepted that damage resulting from reactive oxygen species (ROS) or oxidative stress plays a role in the seed aging process. ROS are highly reactive and may modify and inactivate proteins, lipids, DNA, and RNA and induce cellular dysfunctions. Plants require high contents of antioxidants such as ascorbate, α-tocopherol, glutathione, and phenolic compounds that can remove ROS as a defense mechanism (Schoner and Heinrich Krause [1990]), and enhance the activities of antioxidant enzymes like superoxide dismutase (SOD), peroxidase (POD), catalase (CAT), and ascorbate peroxidase (APX) (Bowler et al. [1992]). If balances are broken between ROS generation and removal in plant cells, oxidant losses occur along with damage to lipid peroxidation and cell membranes (Larson [1995]).

In the present study, the wintering of shattered seeds of weedy rice on paddy fields was compared with cultivated rice, and seed deterioration and related characteristics concerning seed wintering are reported.

## Results

**Figure 1:**
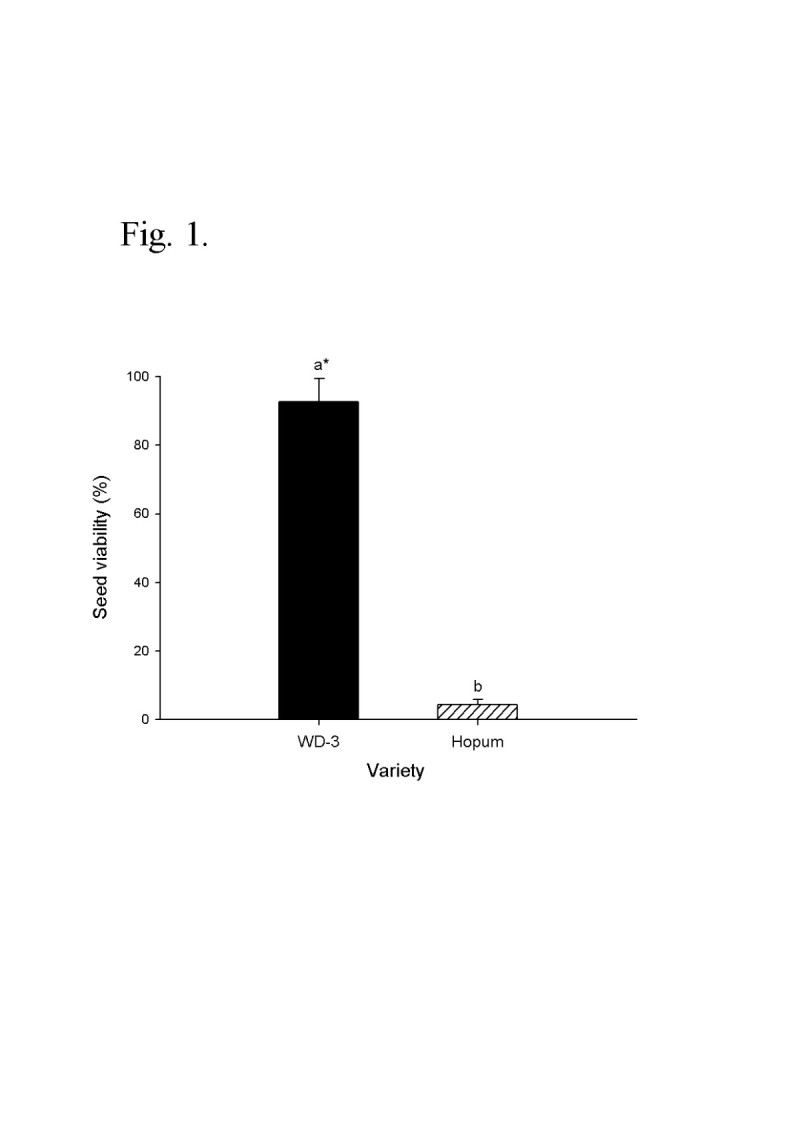
**Seed viability of weedy rice (WD-3) and cultivated rice (Hopum) after wintering on the surface of a paddy field.** After harvesting Hopum, natural shattering weedy rice (WD-3) and artificially shattering cultivated rice (Hopun) were placed on the soil surface at 3 spots of the paddy field under wire net protection during winter from November 2008 to April 2009. Germination was tested at 25°C for 14 days. *Different letters on the bars indicate significant difference of means at α = 0.01 by *t*-test.

**Figure 2:**
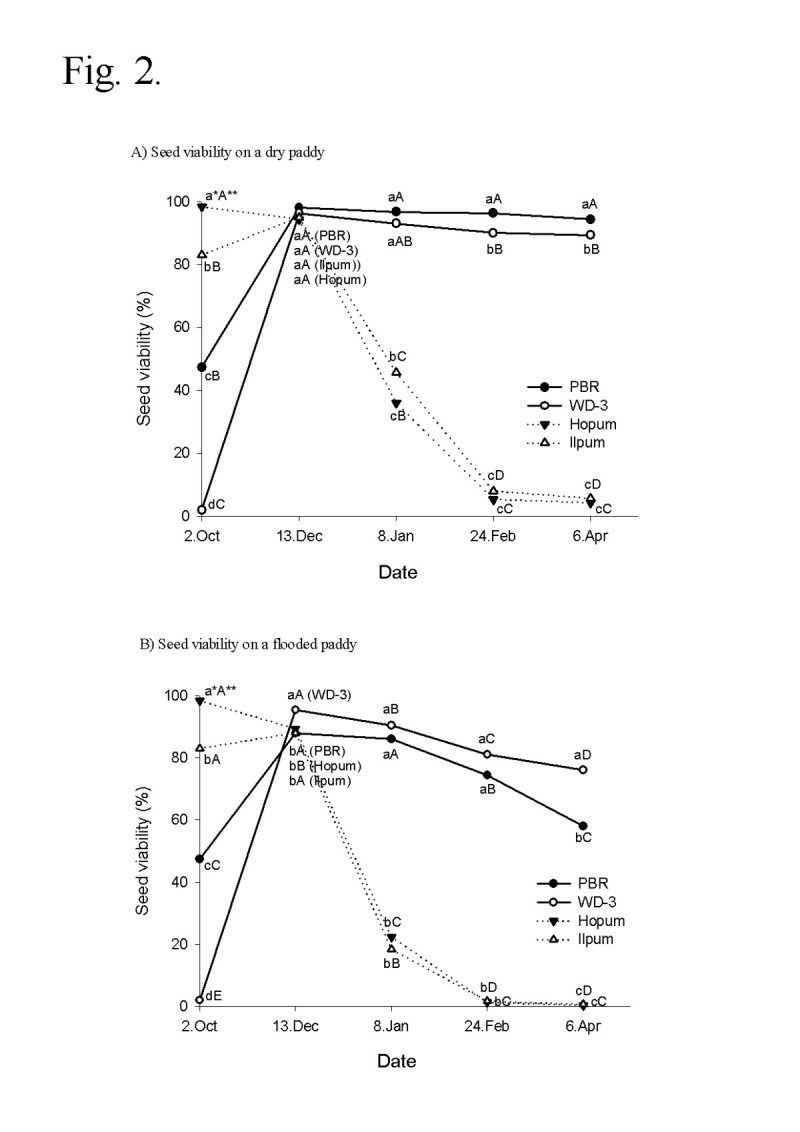
**Time course of seed viability during wintering on dry (A) and flooded (B) paddy conditions in weedy rice (PBR, WD-3) and cultivated rice (Hopum, Ilpum).** The seeds of 4 varieties (200 g each) were kept on the paddy from November 2009 to April 2010. One was a dry paddy(A) in the same way as the first wintering test and the other was a flooded paddy(B) in a plastic box (41.0 × 24.5 × 15.0 cm) with 10 cm depth of paddy soil (silty clay loam). During wintering, the seeds were sampled 5 times for a viability test. *Different small letters in a same date (for vertical comparison) indicate significant difference of means at α = 0.05 by Duncan’s Multiple Range Test. **Different capital letters in the same variety (for horizontal comparison) indicate significant difference of means at α = 0.05 by DMRT.

Seed viability of weedy rice (WD-3) and cultivated rice (Hopum) after a wintering test on the surface of a paddy field is shown in Figure 1. The viability of wintered seeds was 92.7% for the weedy rice and just 4.3% for the cultivated rice. Wintering characteristics of weedy rice in the winter period may vary depending on the moisture state of the paddy surface. Therefore, the second field experiment for seed wintering was carried out in a flooded paddy as well as in a dry paddy field. The results were almost identical to the first year experiment except that the seed viabilities significantly decreased in the flooded paddy as compared with the dry paddy field for both weedy rice (WD-3, PBR) and cultivated rice (Hopum, Ilpum). As shown in Figure 2, the seed viability of cultivated rice rapidly decreased from December to February regardless of paddy conditions and the final average viabilities after wintering were 5.0% (4.3% in Hopum, 5.7% in Ilpum) in the dry paddy and 0.5% (0.3% in Hopum, 0.7% in Ilpum) in the flooded paddy. For weedy rice, the viability was maintained over 90% in the dry paddy, but decreased slightly during winter to a mean of 61% (WD-3: 76%, PBR: 58%) in the flooded paddy field. At the early stage of wintering, the germination percentage of weedy rice was much lower overall than of cultivated rice. This may be because weedy rice had primary seed dormancy (Bewley and Black [1982]; Cohn and Hughes [1981]), and generally exhibited stronger dormancy than cultivated rice (Gu et al. [2003]). In considering the relationship of seed dormancy to wintering, a second area of interest is whether dormancy increases the lifespan of seeds. In this result, the dormancy of all seeds disappeared in December regardless of its intensity and did not affect the resistant to wintering and freezing stress. Roberts ([1972]) reviewed on the subject and concluded that available evidence was not sufficient to establish even a causal relationship. However, this does not exclude the possibility that the lifespan of dormant seeds of native or wild plants buried in the soil under natural conditions may be longer than the lifespan of non-dormant seeds (Justice and Bass [1978]).

**Figure 3:**
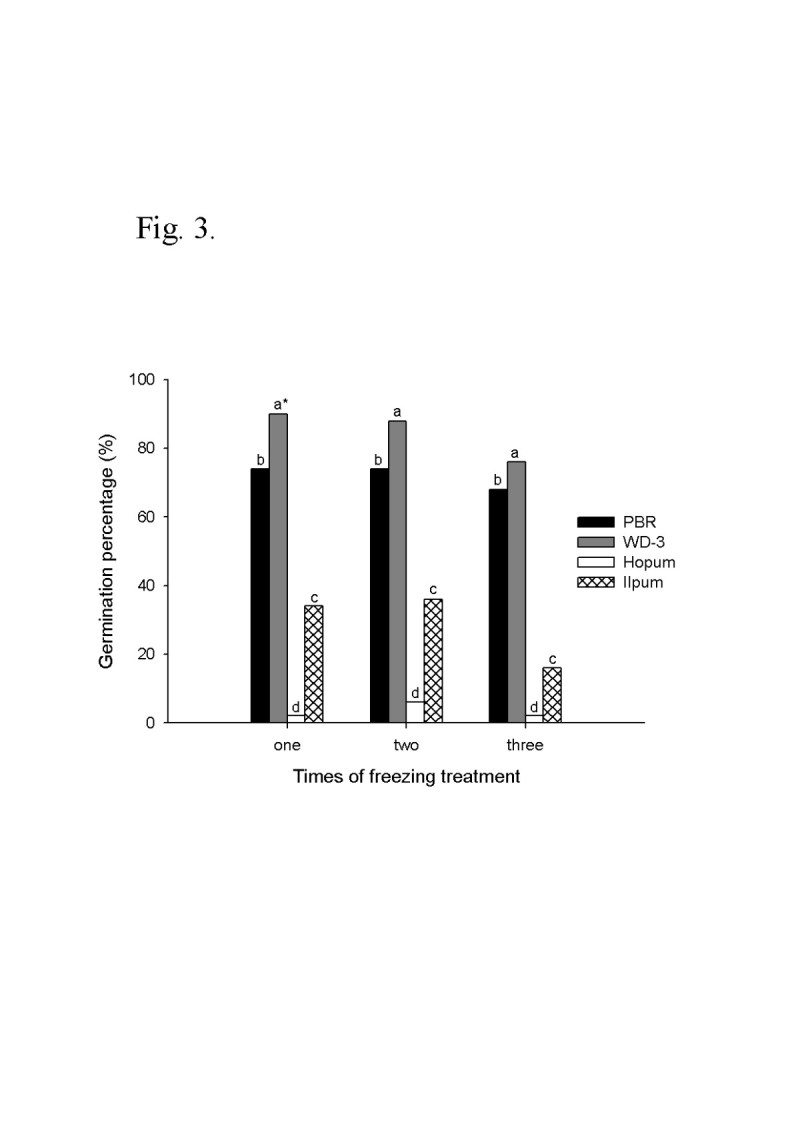
**Germination percentage of seeds after a number of freezing treatments in weedy rice (PBR, WD-3) and cultivated rice (Hopum, Ilpum).** The seeds were imbibed at 4°C for 24 hours and drained on paper towel. Next, they were frozen at −10°C for 24 hours, and then thawed at 4°C for 24 hours. This was one cycle of freezing treatment. After 1 to 3 cycles of treatment, germination was tested at 25°C for 14 days. . *Different letters on the bars in a treatment indicate significant difference of means at α = 0.01 by Duncan’s Multiple Range Test.

Viability of seed wintered on the paddy surface may be affected by temperature changes. In particular, subzero temperatures after rain and snow may freeze the seeds and cause them to die. Therefore, in order to winter safely under subzero temperature, seeds should have resistance to freezing injury for not losing its viability even under soaking conditions. Figure 3 shows seed viability after a number of freezing treatments (1–3 cycles). Seed viability values on average of three treatments of freezing were about 78% (PBR: 72%, WD-3: 85%) in weedy rice and 16% (Hopum: 3%, Ilpum: 29%) in cultivated rice. The results indicated that the weedy rice had much higher resistance than the cultivated rice varieties, and there were also considerable variations among accessions regardless of the number of freezing treatments.

**Figure 4:**
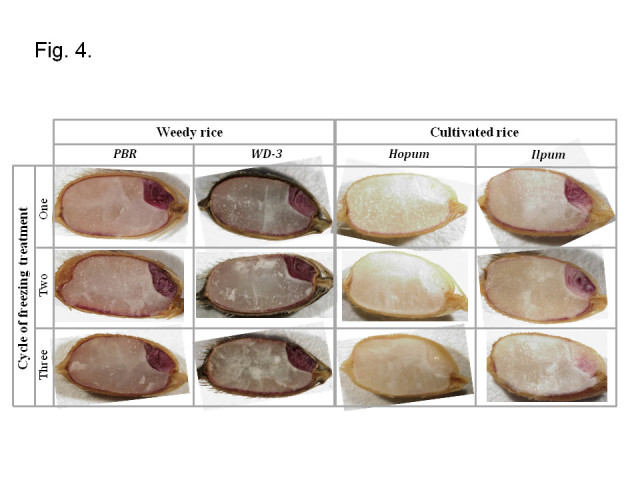
**Tetrazolium test of seeds after a number of freezing treatments in weedy rice (PBR, WD-3) and cultivated rice (Hopum, Ilpum).** The method of freezing treatment was the same as for Figure 3.

The deterioration of seed tissue induced by freezing treatment was observed by the tetrazolium test as shown in Figure 4. The frequency of dark purple stained seeds matched up with the freezing resistance of 4 accessions, as shown in Figure 3. In most seeds of weedy rice (PBR and WD-3), whole embryo tissue and the aleuronic layer were stained dark purple. In the cultivated rice, however, the seeds of Hopum were not completely stained, but those of Ilpum were partially stained, which results indicated that freezing resistance of Ilpum was higher than that of Hopum, and there were variation of freezing resistance among individual seeds in a variety and among rice varieties. Some seeds partially damaged were stained in a light pink at overall tissue such as radicle, plumule, scutellum in embryo and aleurone layer in endosperm. In the deterioration process of seeds under subzero temperature, there was no special tissue structure initiating deterioration.

**Figure 5:**
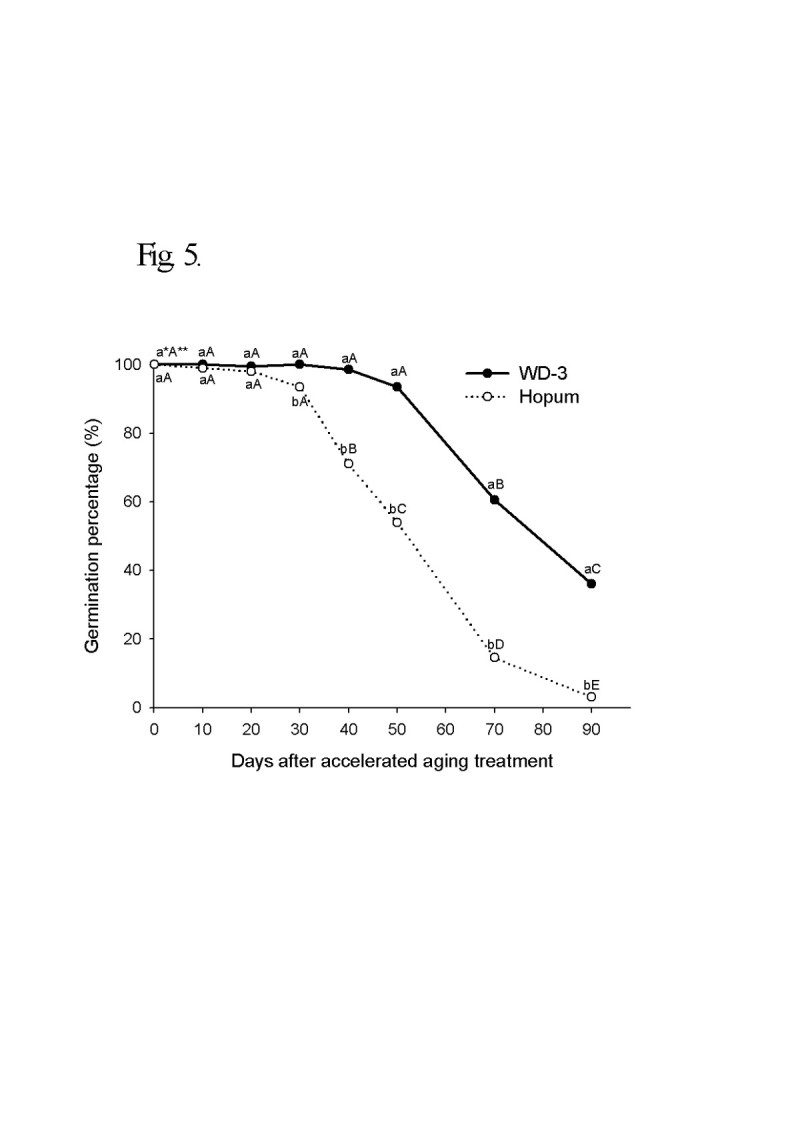
**Germination percentage of rice seeds during accelerated aging treatment.** For the accelerated aging treatment, the seeds were kept soaking in distilled water at 4°C for 90 days. The seeds were sampled and tested at 10 day intervals. Germination tests were carried out with 4 replicates of 100 seeds at 25°C for 14 days. *Different small letters in a same date (for vertical comparison) indicate significant difference of means at α = 0.05 by *t*-test. **Different capital letters in a same variety (for horizontal comparison) indicate significant difference of means at α = 0.05 by Duncan’s Multiple Range Test.

During the winter season, shattered seeds on the paddy surface tended to remain wet at low temperature. Therefore, an accelerated aging test was set up by soaking the seeds at a low temperature (4°C). In this condition, the change of seed viability, physiochemical properties including protein content, fat acidity, and some antioxidant enzyme activities were observed at 10 day intervals during treatment. As shown in Figure 5, the seed viability of weedy rice under the accelerated aging condition started to fall off 50 days after treatment and reached approximately 40% 90 days after treatment. In the cultivated rice, it started to fall off 20 days after treatment, and reached 4% 90 days after treatment.

**Figure 6:**
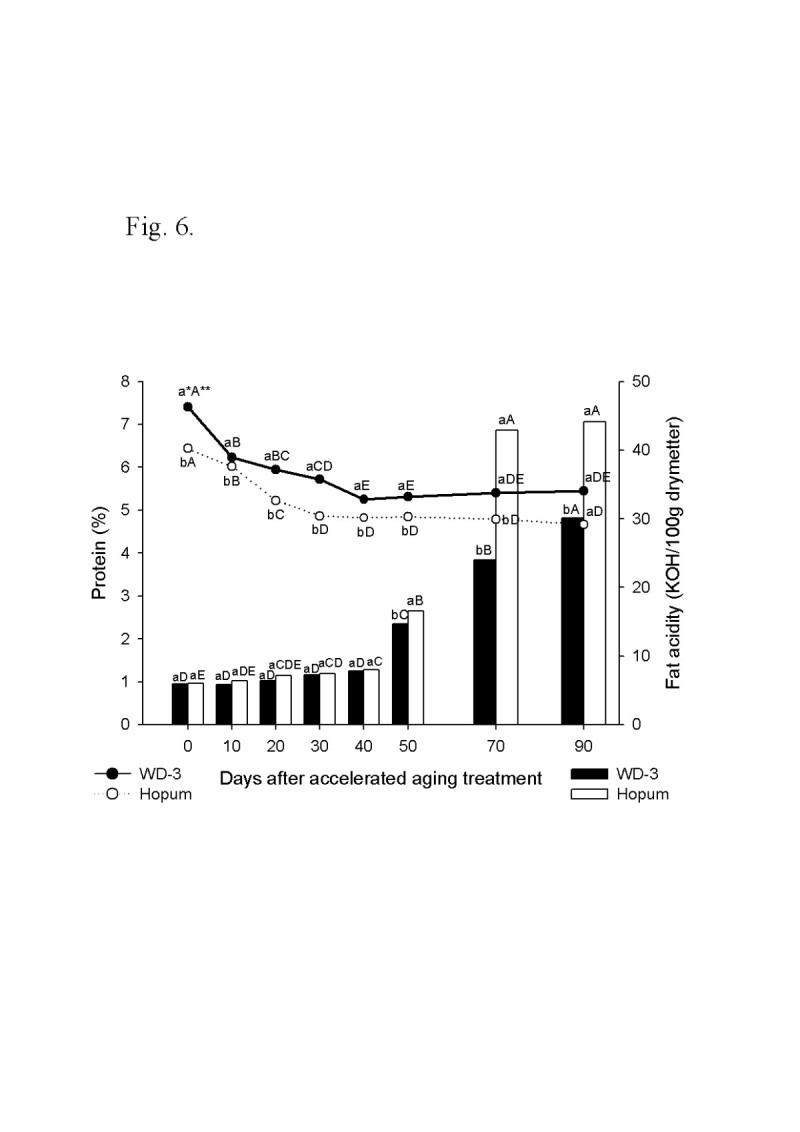
**Time course of protein content and fat acidity during accelerated aging treatment for 90 days.** The accelerated aging treatment was the same as for Figure 5. * Different small letters in a same date (for vertical comparison) indicate significant difference of means at α = 0.05 by *t*-test. ** Different capital letters in a same variety (for horizontal comparison) indicate significant difference of means at α = 0.05 by Duncan’s Multiple Range Test.

The time courses of protein content and fat acidity in seeds during accelerated aging treatment are shown in Figure 6. The protein contents of WD-3 and Hopum, which were 7.41% and 6.44% before treatment, respectively, significantly decreased to 5.25% and 4.82% 40 days after treatment, and then maintained at this amount until 90 days after treatment without significant change.

In the case of fat acidity, the initial values were 6.50 KOH mg/100 g in the weedy rice and 6.59 KOH mg/100 g in the cultivated rice, which were the same between the two varieties. Until 40 days after treatment, there were no significant changes of fat acidity, but there was a significant increase and difference between weedy rice and cultivated rice 50 days after treatment. Fat acidities of weedy rice and cultivated rice were 42.9 KOH mg/100 g and 23.9 KOH mg/100 g at day 70, and 44.1 KOH mg/100 g and 30.0 KOH mg/100 g at day 90, respectively.

**Figure 7:**
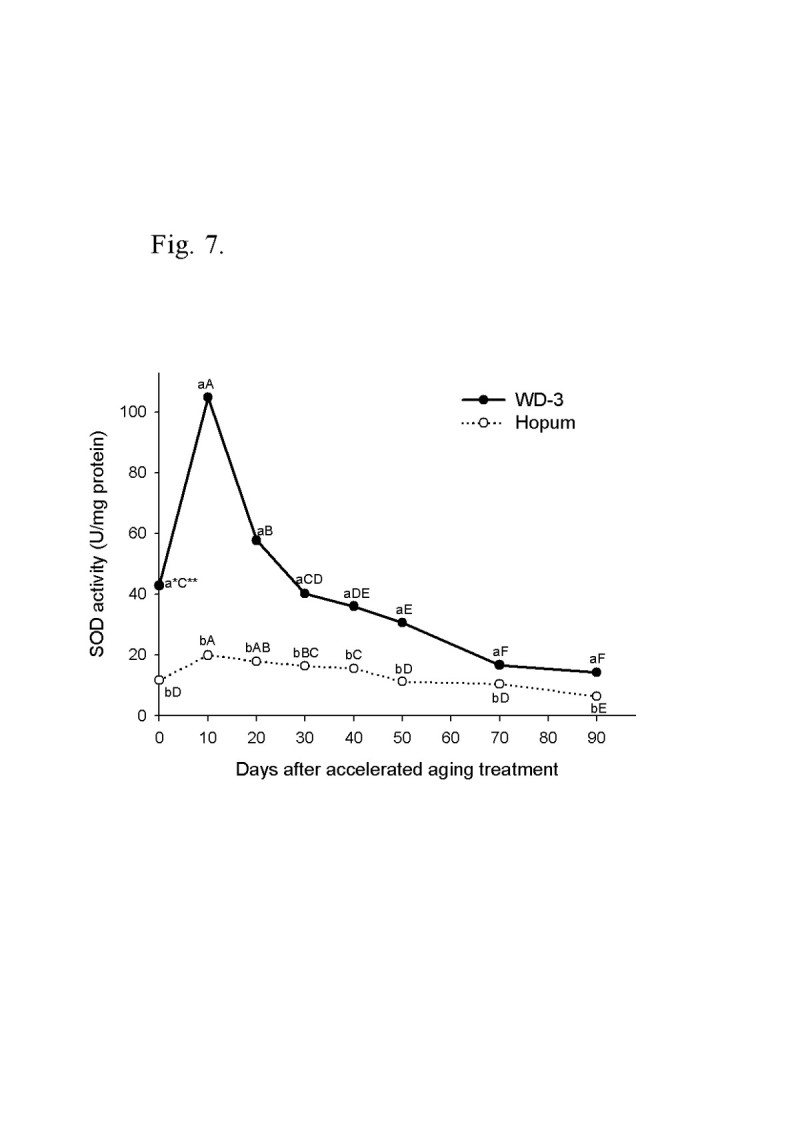
**Time course of superoxide dismutase activity during accelerated aging treatment for 90 days.** The accelerated aging treatment was the same as for Figure 5. * Different small letters in a same date (for vertical comparison) indicate significant difference of means at α = 0.05 by *t*-test. ** Different capital letters in a same variety (for horizontal comparison) indicate significant difference of means at α = 0.05 by Duncan’s Multiple Range Test.

The change of superoxide dismutase (SOD) activity during accelerated aging treatment is shown in Figure 7. The initial values of SOD in the seeds were 42.9 units/mg protein in WD-3 and 11.7 units/mg protein in Hopum. Following accelerated aging treatment, SOD activities of seeds increased for 10 days to 104.9 units/mg protein in WD-3 and to 19.9 units/mg protein in Hopum. After peak SOD activity, they decreased in both weedy and cultivated rice until the end of treatment. The change of SOD activity of weedy rice seed rapidly increased and decreased compared to Hopum, but it was maintained at higher levels than that of Hopum. At day 90 of accelerated aging treatment, the SOD activities were 14.3 units/mg protein in weedy rice and 6.4 units/mg protein in cultivated rice. The results of SOD activity changes during accelerated aging suggested that the response of weedy rice seed to environmental stress was more sensitive than cultivated rice.

**Figure 8:**
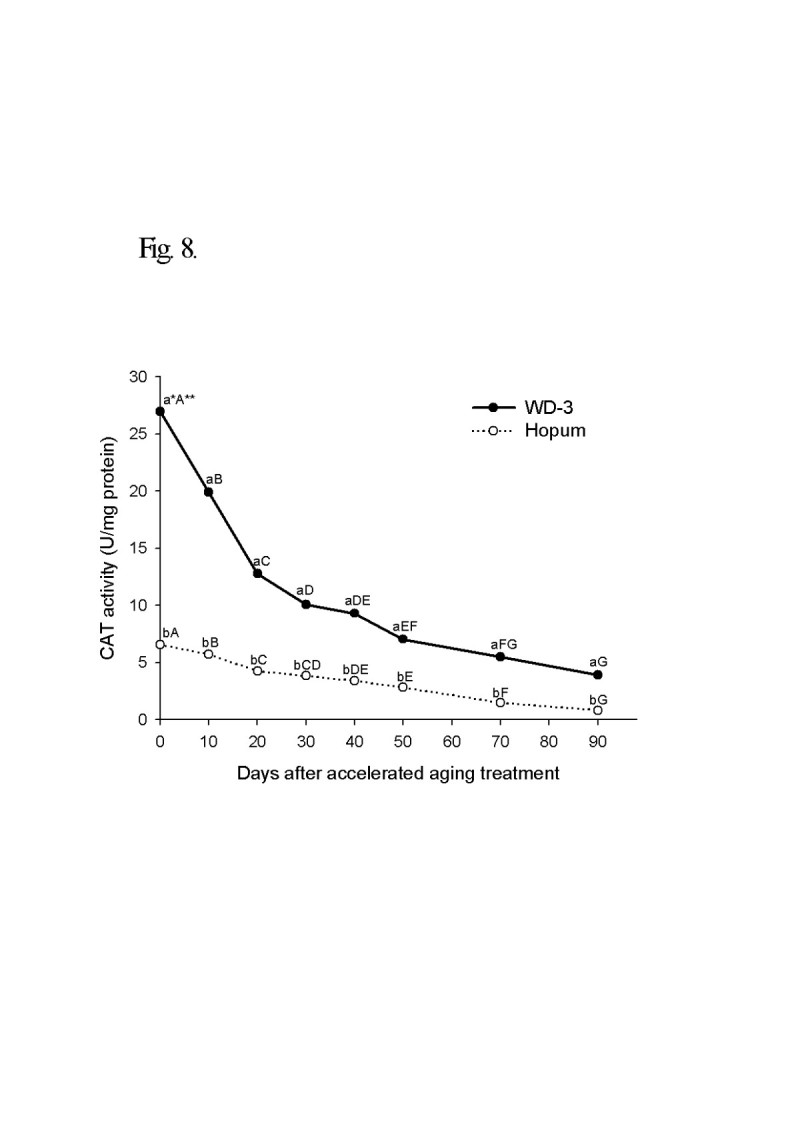
**Time course of catalase activity during accelerated aging treatment for 90 days.** The accelerated aging treatment was the same as for Figure 5. * Different small letters in a same date (for vertical comparison) indicate significant difference of means at α = 0.05 by *t*-test. ** Different capital letters in a same variety (for horizontal comparison) indicate significant difference of means at α = 0.05 by Duncan’s Multiple Range Test.

Change in catalase (CAT) activity in the seeds is shown in Figure 8. The initial CAT activity of WD-3 was much higher than that of Hopum. They were 26.9 units/mg protein in weedy rice and 6.6 units/mg protein in cultivated rice. After accelerated aging treatment, CAT activities gradually decreased in both weedy and cultivated rice to 3.9 units/mg protein in weedy rice and 0.8 units/mg protein in cultivated rice at day 90 of accelerated aging treatment. The CAT activity of weedy rice seed persisted at a significantly higher level than that of cultivated rice seed during accelerated aging treatment.

**Figure 9:**
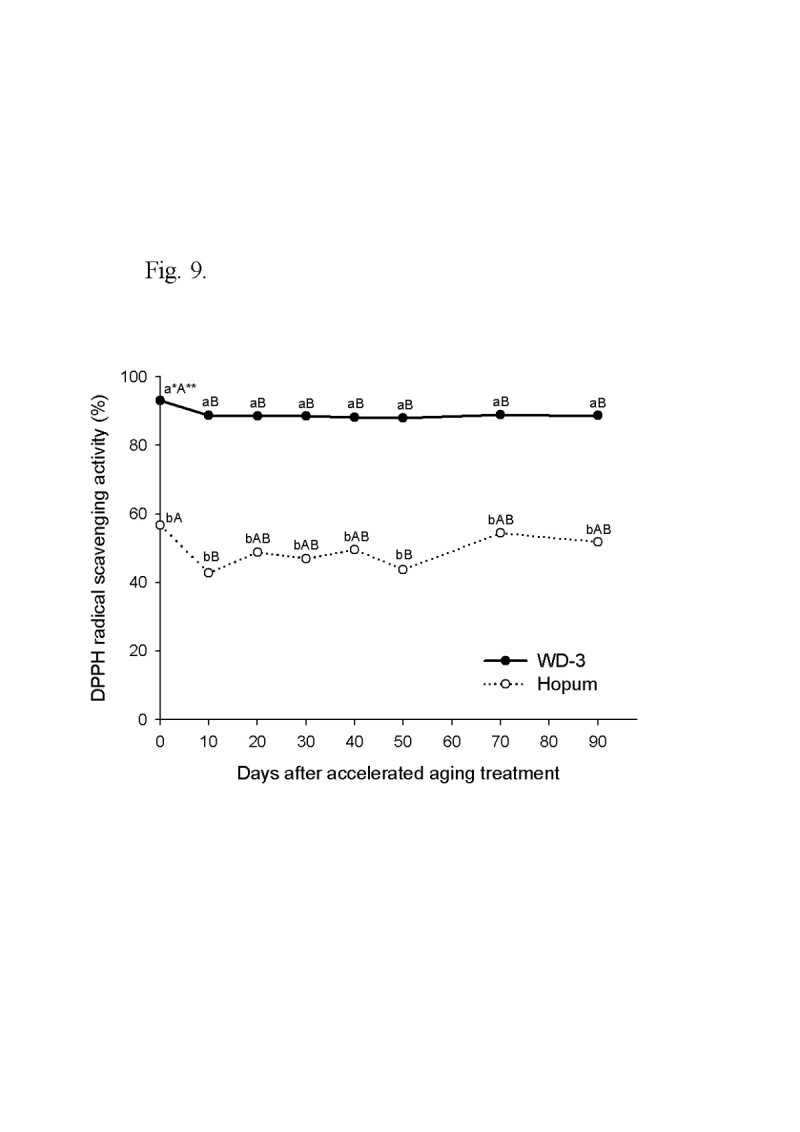
**Time course of DPPH radical scavenging activity during accelerated aging treatment for 90 days.** The accelerated aging treatment was the same as for Figure 5. * Different small letters in a same date (for vertical comparison) indicate significant difference of means at α = 0.05 by *t*-test. ** Different capital letters in a same variety (for horizontal comparison) indicate significant difference of means at α = 0.05 by Duncan’s Multiple Range Test.

Free radical scavenging of phytochemical substances in seeds is an important and generally accepted mechanism for antioxidants inhibiting oxidation (Bandoniene and Murkovic [2002]). The results of seed antioxidant activity measurement during accelerated aging using DPPH is shown in Figure 9. The initial radical elimination activity of WD-3 seed was 93.0%, which was much higher than the 56.6% of Hopum. This persisted during accelerated aging for 90 days without major changes, with approximately 90% in weedy rice and 50% in cultivated rice.

## Discussion

In this study, the wintering rate of weedy rice seeds was about 90% on the dry paddy and 60–80% on the flooded paddy, which was much higher than that of cultivated rice (below 5%). This result was in agreement with several reports about the high germination percentage of weedy rice seeds placed in shallow depths or on the soil surface of paddy fields in the winter (Chung et al. [2000]; Fogliatto et al. [2010]; Ko [2001]; Noldin et al. [2006]; Seong et al. [2004]). Therefore, if a seed of weedy rice spread, it could occur every year and exponentially increase in the rice field. Fogliatto et al. ([2011]) highlighted the significant efficacy of winter flooding in reducing weedy rice infestations in paddy fields. This report is meaningful because the practice showed a significant reduction of weedy rice seed density compared to overwintering under dry conditions, and also winter flooding could favor the decay of seeds of several weeds including weedy rice (Nelms and Twedt [1996]). However, results reported here indicated more than 60% of weedy rice seeds could overwinter under flood conditions. Therefore, winter flooding may not be completely effective but may be an accompanying method to control weedy rice in Korea.

In the winter season, the major factors affecting seed longevity in the field may include precipitation and subzero temperature, as shown in meteorological data in Table 1. When the plant is being frozen gradually, moisture leaves the cell to the outside of the membrane and forms ice crystals causing mechanical damage (Kim [2010]). In the case of seed, if its moisture content is high, it is easily deteriorated in above freezing temperature owing to an increase in respiration and metabolism, and may get damaged in the winter or die at subzero temperature (Baek et al. [2000]).

**Table 1:** **Meteorological data on temperature and precipitation in Jeonju city during the seed wintering test in the 2008/2009 and 2009/2010 seasons**

Year	Month	Temperature (°C)			Days of subzero temperature	Precipitation (mm)	Precipitation days
		Mean	Maximum	Minimum			
2008	Nov.	8.5	14.0	3.8	6	1.4	9
	Dec.	3.1	7.9	−1.7	20	1.1	5
2009	Jan.	−0.4	5.9	−5.1	29	0.9	11
	Feb.	4.2	9.5	−0.6	15	6.0	6
	Mar.	7.0	13.4	1.5	13	4.6	10
	Apr.	12.6	20.2	6.3	1	3.3	5
2009	Nov.	8.6	13.5	4.3	7	2.1	9
	Dec.	1.3	5.7	−2.9	24	2.7	11
2010	Jan.	−0.8	4.1	−5.3	30	3.0	8
	Feb.	3.2	7.7	−0.8	16	7.4	10
	Mar.	6.3	10.9	1.6	9	4.4	14
	Apr.	10.3	16.2	4.8	5	7.0	11

As shown in Figure 3, the freezing resistance of weedy rice seed was much higher than that of cultivated rice. Weedy rice surviving semi-wildly in rice fields is known to show a high level of adaptability to environmental stress (Chung [2010]; Chung and Ahn [2005]; Suh [2003]). The weedy seeds that shatter on the surface of paddy fields in the autumn should have resistance to freezing in the winter to regenerate in the following germinating season, although there was no report on the freezing resistance of weedy rice seeds. In plants with freezing resistance, antifreeze proteins block the growth of ice crystals in the outside spaces of cells in the tissue, thus preventing cell damage derived from the repetition of freezing and thawing, and also have functions in transforming the generation of ice crystals by adhering to surfaces of ice (DeVries [1986]; Jeong [2009]; Yoo and Hwang [2000]). In this way, antifreeze protein accumulates in the tissue in case of cold acclimation, and thus makes the plant not freeze to death even in freezing temperature. The tetrazolium test in Figure 4 showed that freezing damage of the cultivated rice began in overall tissue of the embryo and aleuronic layer of seed. But, in the weedy rice seeds after freezing treatment, those seed tissues were stained by tetrazolium solution to dark purple to show freezing resistance. Therefore, one hypothesis for a freeze resistance mechanism of weedy rice is that anti-freeze proteins accumulate in the embryo and aleuronic layer of the seed.

The accelerated aging test has been recognized as an accurate indicator of seed vigor and longevity, and may be generally induced by high temperature and high humidity (Hsua et al. [2003]). The deterioration of seeds wintering on the field surface in temperate regions could be affected by high moisture content at low temperature as discussed earlier, thus accelerated aging can be induced by soaking at low temperature (4°C). The seeds deteriorated rapidly under this accelerated aging method, and there was a large difference in deterioration rate between weedy and cultivated rice. The viabilities of seeds after 90 days of accelerated aging were 4% in cultivated rice and 40% in weedy rice.

The deleterious effect of accelerated aging on the longevity of seeds was associated with the damage occurring to lipid, nucleic acid, and protein owing to oxidative stresses (Cakmak and Horst [1991]; Fujikura and Karssen [1995]; Hsua et al. [2003]; Ito et al. [1999]; McDonald [1992]; Pilar [1998]; Shewfelt and Purvis [1995]; Vartapetian and Jackson [1997]; Yan et al. [1996]). In the accelerated aging test, the difference of protein contents and fat acidities in seeds between weedy and cultivated rice indicated that weedy rice had a higher protective system to aging stress than the cultivated rice.

Scandalios ([1993]) reported that SOD and CAT were effective antioxidant enzymes for decreasing oxidant damage of cells by oxidative stress. The activity of SOD before and after the accelerated aging treatment for 90 days in weedy rice was much higher than in cultivated rice. However, the SOD activities of seeds rapidly increased and decreased with the peak occurring 10 days after treatment. SOD activity decreased if stresses persisted for a long time or excessive stresses were added (Kim and Lee [1992]). CAT is considered a primary enzymatic defense against oxidative stress induced by senescence, chilling, dehydration, osmotic stress, wounding, paraquat, ozone and heavy metals (Kibinza et al. [2011]). Shon et al. ([2008]) reported that CAT activity had exhibited a decreasing tendency upon submerging stress in young rice seedlings, and appeared as the most sensitive enzyme to stress among antioxidant components, regardless of whether it was in seeds. Similarly, CAT activity was reduced after treatment in both the weedy and cultivated rice, where the activity of weedy rice persisted at a higher rate than the cultivated rice. Tanida ([1996]) reported that CAT activity of the embryo in the seed soaked at low temperature had positive correlation with germination percentage after treatment. High SOD and CAT activities, as well as radial scavenging activity were considered to increase the vitality of seeds from excessive stress damage in soaking at low temperature.

## Conclusion

In conclusion, the superior ability of seed wintering in weedy rice was based on the freezing resistance of embryo tissue and the ability of antioxidant enzymes and photochemicals to reduce seed deterioration.

## Methods

### Plant materials

Two japonica weedy rice, PBR and WD-3, and two Korean bred cultivars (japonica), Hopum and Ilpum, were used in this experiment. Prior to the laboratory-based experiment, they were regenerated in the experimental field in Chonbuk National University to obtain fresh seeds. The weedy rice were harvested at 30 days after heading, and the cultivated rice were harvested at 50 days after heading. Seeds were air dried until seed moisture content (SMC) became less than 14%. The SMC was measured by oven-drying method. The seeds were sealed in plastic bags, and stored at 4°C until use for about 7–8 months. When they were used, the SMCs were about 12% regardless of varieties.

### Seed wintering test on the paddy field

In the first wintering experiment, Hopum, a bred cultivar, was cultivated in the dry paddy field where the weedy rice (WD-3) occurred at a rate of more than 500 plants per m^2^. After harvesting Hopum, naturally shattered weedy rice (WD-3) seeds and artificially shattered cultivated rice (Hopum) seeds were placed on the soil surface at 3 spots of the paddy field under wire net protection during winter from November 2008 to April 2009. Germination tests of seeds collected at 3 spots after wintering were conducted at 25°C for 14 days using 4 replicates of 100 seeds each in accordance with the International Rules for Seed Testing for *Oryza sativa* (ISTA [2009]).

In the second wintering experiment, two weedy rice varieties (WD-3 and PBR) and two Korean cultivars (Hopum and Ilpum) were tested. The seeds of 4 varieties (200 g each) were kept on the paddy from November 2009 to April 2010. The paddy conditions were maintained as two types during winter. One was a dry paddy as was used for the first wintering test and the other was a flooded paddy simulated by plastic boxes (41.0 × 24.5 × 15.0 cm) filled with 10 cm depth of paddy soil (silty clay loam) and superabundant water. During wintering, the seeds (400 grains each from three replicates) were sampled 5 times for viability tests (ISTA [2009]). The meteorological data on temperature, subzero temperature days, precipitation, and precipitation days during the wintering test for 2008/2009 and 2009/2010 is shown in Table 1.

### Seed freezing test

To investigate the resistance of seeds under subzero temperature, the weedy (PBR, WD-3) and cultivated rice (Hopum, Ilpum) were assigned to freezing treatment as follows; the seeds were frozen at −10°C for 24 hours after imbibing at 4°C for 24 hours and then thawed at 4°C for 24 hours. The freezing method was repeated 1 to 3 times, and then the viability of the seeds was tested as stated above in seed wintering test. After freezing treatment, a tetrazolium test was also carried out to investigate the state of seed tissues. Soon after freezing treatment, seeds were kept at 25°C for 15–18 hours. Following that, seeds and embryos were cut vertically using a razor blade, placed in 1% tetrazolium solution (pH 6.5–7.5) at 30°C for 2 hours, and the staining patterns were observed under a dissecting microscope.

### Seed viability and physiochemical test under accelerated aging treatment

To determine the deterioration of soaked seed at low temperature, the seeds of weedy rice (WD-3) and cultivated rice (Hopum) were kept soaking in distilled water at 4°C for 90 days as an accelerated aging treatment, and viability and physicochemical changes of seeds were observed at 10 day intervals. During accelerated aging treatment with three replicates, a 100 g of seeds per replicate was sampled at each sampling time. Among the sampled seeds, 400 grains per replicate was used for the viability test and the rest was ground with liquid nitrogen to powder by auto-mill (TK-AM5, Tokken Inc.) and then dried by freeze dryer (Clean Vac 8, BioTron). The powder was used to analyze protein content, fat acidity, superoxide dismutase (SOD) activity, catalase (CAT) activity and 2,2-diphenyl-2-picrylhydrazyl (DPPH) radical scavenging activity. A germination test was conducted as for the wintering test with four replicates.

Protein content was measured in three replicates by the micro-Kjeldahl method (AOAC [1990]). Seed powder (0.2 g per replicate) was weighed into the Kjeldahl flask. Sulfuric acid and a catalyst (Kjeltabs auto: 1.5 g K_2_SO_4_, 7.5 mg Se) were added to the flask and the mixture was heated to 420 °C for 1 hour. Total nitrogen of each protein fraction was determined by the automatic Kjeldahl nitrogen analyzer (Kjeltec 2400 Analyzer Unit, Foss Tecator) and protein content was calculated using a conversion factor of 5.95, as shown in the equation below:

1 $$\text{Protein}\left(\%\right)=\text{N}\left(\%\right)\times 5.95$$

Fat acidity (KOH mg/100 g) was analyzed in three replicates by the AOAC ([1970]) method. It was determined by the amount of KOH required to reduce free fat acid included in 100 g of dry material. A total of 10 g of seed powder was placed in a 100 ml flask followed by addition of 50 ml of benzene and 30 min. agitation (150 rpm, 25°C). The mixed solution was filtered through filter paper (Adventec No. 2, 90 mm), and 15 ml of the solution was put in a 100 ml flask and treated with 15 ml phenolphthalein melted in ethyl alcohol. The solution was finally titrated with 0.0045 N-KOH. Ending point of the reaction was determined when color of the solution turned pink, which was the standard color. Tests of the blank and standard varieties were carried out at the same time. Fat acidity was calculated by the following formula (AOAC [1970]):

2 $$\text{Fat}\text{acidity}\text{value}=\left(\text{T}-\text{B}\right)\times 8.33\times 100/\left(100-\text{W}\right)$$

T : amount of KOH (ml) used in sample

B : amount of KOH (ml) used in blank

W : moisture content of sample

8.33 : conversion factor according to sample amount and KOH concentration

For analysis of SOD and CAT activity, 1 g of seed powder was homogenized with 5 ml 0.1 M potassium phosphate buffer (pH 7.0) contains 1 mM EDTA and 1 mM DMSO. The homogenized samples were centrifuged at 30,000 × *g* for 20 min. at 4°C. The supernatant was stored at −80°C and used as a crude enzyme extract (Shon et al. [2008]). For SOD activity test by the nitro blue tetrazolium (NBT) reduction method (Beyer [1987]; Shon et al. [2008]), 0.1 ml of 400 mM methionine, 0.1 ml of 2.5 mM NBT and 0.3 ml of 1 mM EDTA were vortexed in the glass test tube at 25°C for 3 min. for temperature balance. Then, 0, 20, 40, and 60 μl of enzyme extracts and 2.45, 2.43, 2.41 and 2.39 ml of 50 mM potassium phosphate buffer (pH 7.8) were added respectively to make final volume of 3 ml of enzyme and potassium phosphate buffer. 50 μl of 120 μM of riboflavin was added to each test tube. The test tubes were softly shaken and placed under fluorescent lamps for 15 min., and absorbance was measured at 560 nm by a spectrophotometer. One unit of SOD was defined as the amount of enzyme necessary to inhibit formation of blue formazan to reduce absorbance to 50% of blank’s.

CAT activity was measured in accordance with Beers and Sizer ([1954]). One ml of 59 mM H_2_O_2_ (Merck’s Supersol) and 1.9 ml of 50 mM potassium phosphate (pH 7.0) were put in the quartz cuvette. 0.1 ml of enzyme extract was added. The absorbance was measured at 240 nm using a spectrophotometer adjusted to 25°C. Finally, decrease in absorbance for 3 min was recorded. One unit decomposes one micromole of H_2_O_2_ per minute at 25°C and pH 7.0. The catalase activity was obtained by the following equation (Beers and Sizer [1954]):

3 $$\text{Units}/\text{mg}\text{protein}=\left(\Delta \text{A}240/\text{min}\times 1000\right)/\left(43.6\times \text{mg}\text{enzyme}/\text{ml}\text{reaction}\text{mixture}\right)$$

DPPH free radical scavenging activity was measured according to Lee et al. ([2008]) based on Blois method ([1958]). A total of 1 g of milled seed sample was extracted by 10 ml methanol at 50°C for 24 hours on a 150 rpm shaker. The filtered sample solution was centrifuged at 12,000× *g* for 20 min, and the supernatant was used for the assay. The reaction mixture containing 1,700 μl of 0.15 mM DPPH in methanol and 300 μl of sample solution was shaken well and incubated for 30 min. at room temperature, and the absorbance of the resulting solution was read at 517 nm. The scavenging activity was determined by comparing the absorbance with that of the control containing equal volumes of DPPH solution and ethanol. The DPPH radical scavenging activity was obtained by the following equation:

4 $$\text{DPPH}\text{radical}\text{scavenging}\text{activity}\left(\%\right)=\left[\left({\text{A}}_{\text{control}}-{\text{A}}_{\text{sample}}\right)/{\text{A}}_{\text{control}}\right]\times 100)$$

### Statistical analysis

For the evaluation of individual treatment means, all collected data from the complete randomized design with three replicates (four replicates in germination test, exceptively), subjected to analysis of variance using Statistical Analysis System (SAS 9.1).

## Authors’ original submitted files for images

Below are the links to the authors’ original submitted files for images. Authors’ original file for figure 1 Authors’ original file for figure 2 Authors’ original file for figure 3 Authors’ original file for figure 4 Authors’ original file for figure 5 Authors’ original file for figure 6 Authors’ original file for figure 7 Authors’ original file for figure 8 Authors’ original file for figure 9
