# Supplementary figures and images for: Seed wintering and deterioration characteristics between weedy and cultivated rice
Source: Rice (N Y). 2012 Aug 17;5:21. doi: 10.1186/1939-8433-5-21 (PMC5520834; doi:10.1186/1939-8433-5-21)

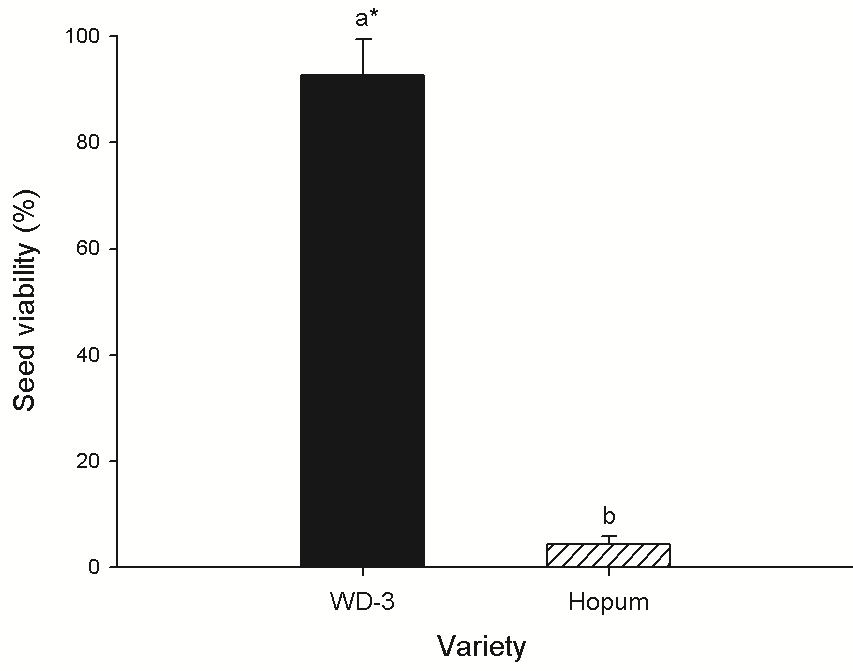

Supplement: Supplementary file 1 — Authors’ original file for figure 1 [file 12284_2012_17_MOESM1_ESM.tiff]

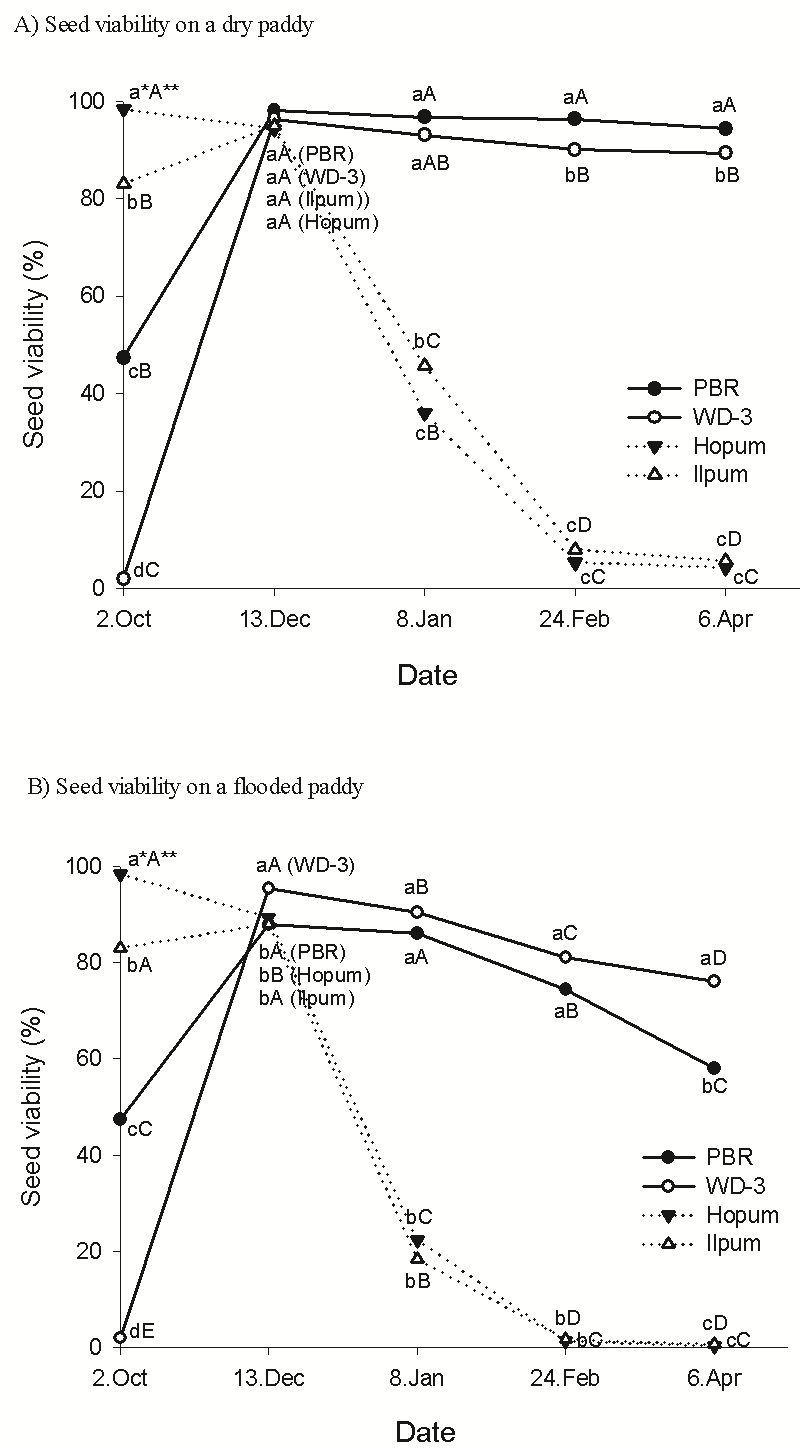

Supplement: Supplementary file 2 — Authors’ original file for figure 2 [file 12284_2012_17_MOESM2_ESM.tiff]

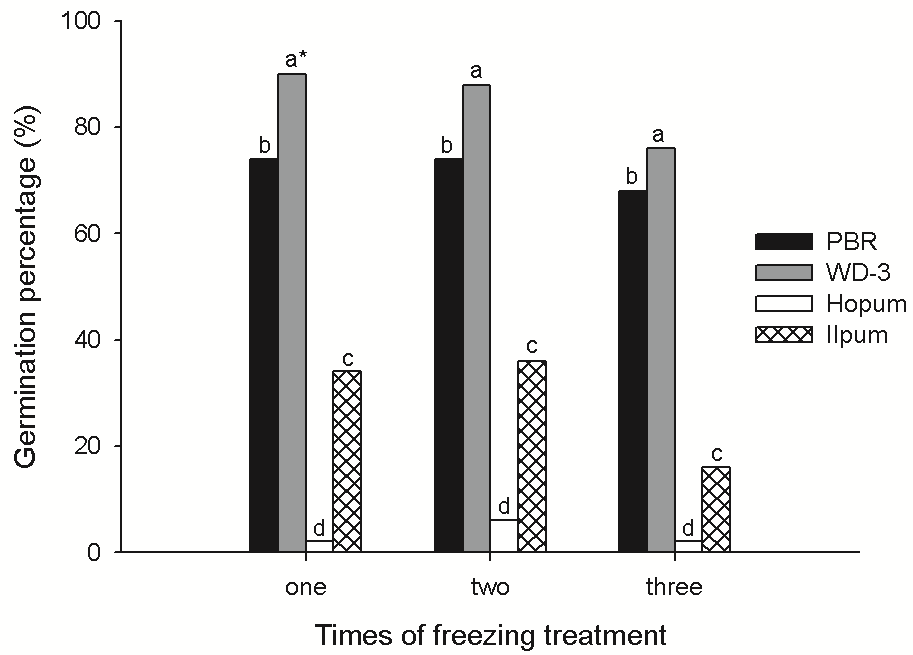

Supplement: Supplementary file 3 — Authors’ original file for figure 3 [file 12284_2012_17_MOESM3_ESM.tiff]

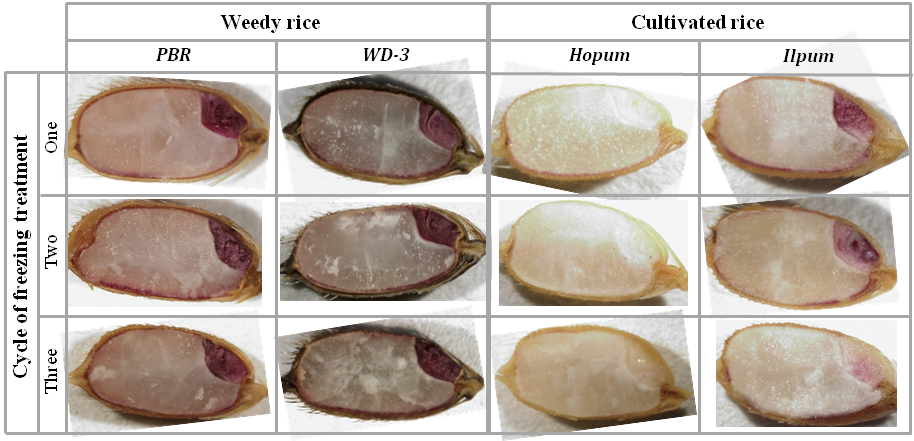

Supplement: Supplementary file 4 — Authors’ original file for figure 4 [file 12284_2012_17_MOESM4_ESM.tiff]

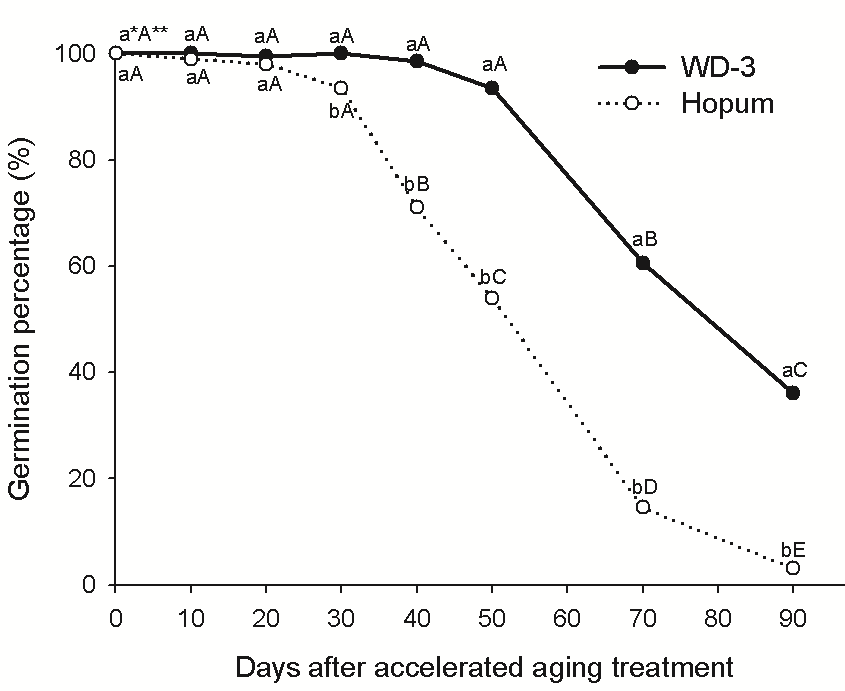

Supplement: Supplementary file 5 — Authors’ original file for figure 5 [file 12284_2012_17_MOESM5_ESM.tiff]

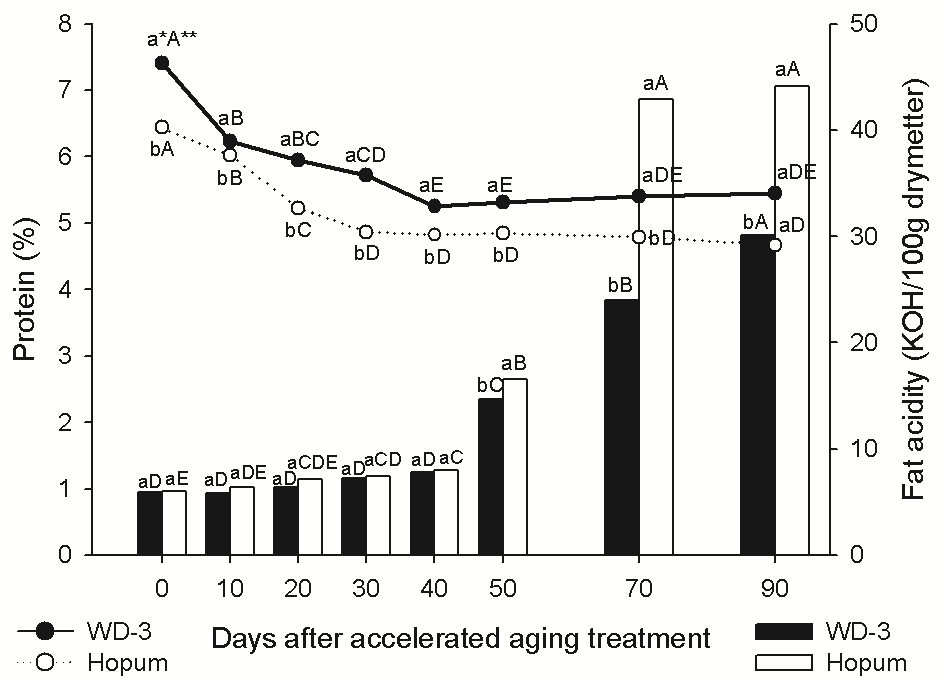

Supplement: Supplementary file 6 — Authors’ original file for figure 6 [file 12284_2012_17_MOESM6_ESM.tiff]

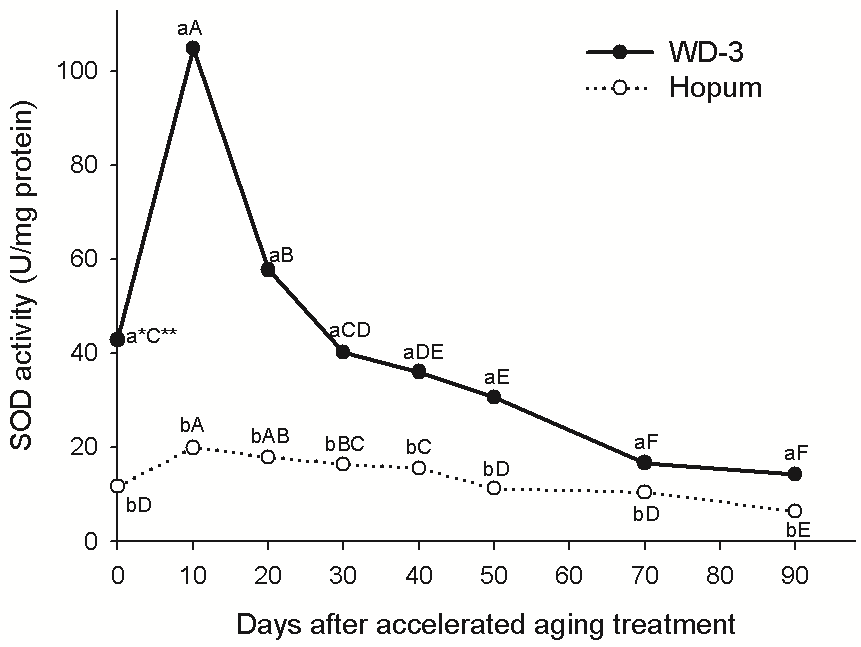

Supplement: Supplementary file 7 — Authors’ original file for figure 7 [file 12284_2012_17_MOESM7_ESM.tiff]

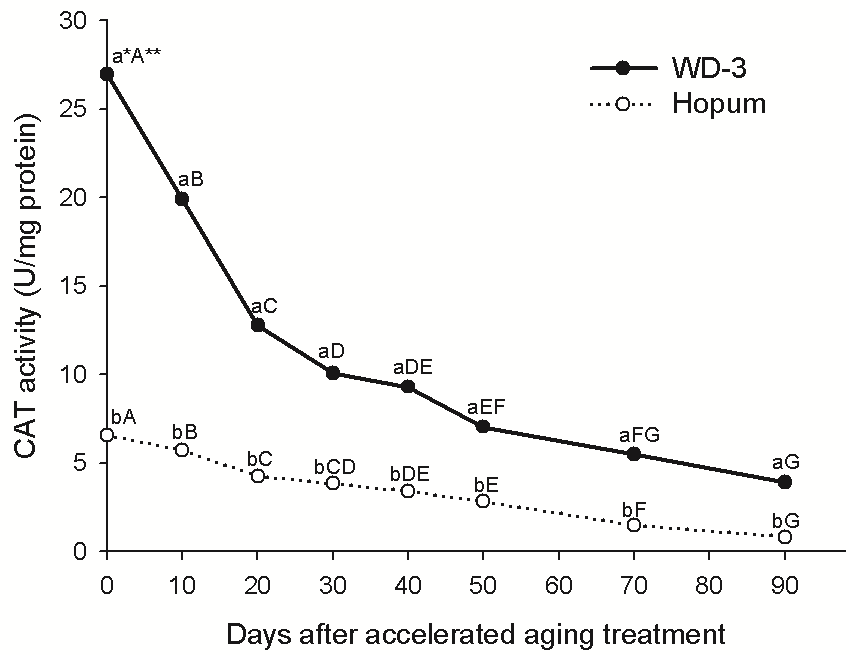

Supplement: Supplementary file 8 — Authors’ original file for figure 8 [file 12284_2012_17_MOESM8_ESM.tiff]

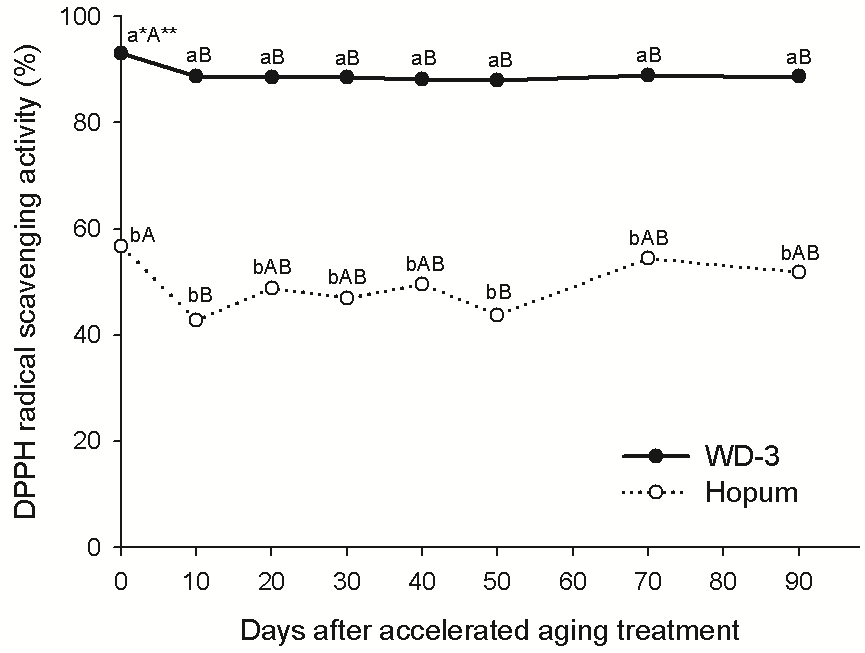

Supplement: Supplementary file 9 — Authors’ original file for figure 9 [file 12284_2012_17_MOESM9_ESM.tiff]
